# Understanding Motor Adaptation in the Transition to Sustained Pain: Protocol for a Longitudinal Experimental Study

**DOI:** 10.2196/99833

**Published:** 2026-06-18

**Authors:** Arnaud Duport, Phivos Phylactou, Gaspard Diotalevi, Dryden Arseneau, Thu Ngoc Minh Pham, Ashley Lowndes, Osvaldo Espin-Garcia, David A Seminowicz, Siobhan M Schabrun

**Affiliations:** 1 The Gray Centre for Mobility and Activity, Lawson Research Institute, London, ON, Canada; 2 School of Physical Therapy, Faculty of Health Sciences, Western University, 1151 Richmond St, London, ON, N6A 5B9, Canada, 1 5196614249; 3 Research Centre on Aging, Sherbrooke, QC, Canada; 4 Department of Mechanical Engineering, Université de Sherbrooke, Sherbrooke, QC, Canada; 5 Graduate Program in Neuroscience, Schulich School of Medicine & Dentistry, Western University, London, ON, Canada; 6 Department of Epidemiology and Biostatistics, Schulich School of Medicine & Dentistry, Western University, London, ON, Canada; 7 Dalla Lana School of Public Health and Department of Statistical Sciences, University of Toronto, Toronto, ON, Canada; 8 Department of Biostatistics and Schroeder Arthritis Institute, University Health Network, Toronto, ON, Canada; 9 Department of Medical Biophysics, Schulich School of Medicine & Dentistry, Western University, London, ON, Canada

**Keywords:** motor adaptation, induced pain, transcranial magnetic stimulation, electroencephalography, cognitive functions, conditioned pain modulation

## Abstract

**Background:**

Chronic pain affects over 30% of the global population and remains a major public health issue due to limited treatment efficacy and the need for mechanism-based, personalized approaches. Motor behavior is theorized to play a role in pain persistence through altered movement patterns, muscle recruitment, and proprioception. While motor behavior is linked to chronic pain, empirical evidence on underlying mechanisms, particularly cortical dynamics, remains scarce.

**Objective:**

This study aimed to investigate longitudinal changes in cortical sensorimotor excitability and their relationship with maladaptive motor behaviors.

**Methods:**

This prospective longitudinal study will follow 150 healthy participants, aged 18‐65 years, recruited from the community, experiencing experimentally induced muscle pain across 4 visits (day 0, day +2, day +4, and 5 days after pain resolution). Pain will be induced using intramuscular injections of nerve growth factor into the extensor carpi radialis brevis muscle. The primary outcome is motor variability assessed during multidimensional wrist movement tasks, and quantified using root-mean-square deviation and muscle synergies (derived from electromyographic recordings of 4 forearm muscles). Secondary outcomes include cortical mechanisms (electroencephalographic peak alpha frequency, transcranial magnetic stimulation mapping, short-interval intracortical inhibition, and intracortical facilitation), sensorimotor integration (evoked potential), cognitive control (multisource interference task), and endogenous pain modulation (conditioned pain modulation). Self-report questionnaires will assess pain intensity and disability (Patient-Rated Tennis Elbow Evaluation, McGill Pain Questionnaire–Short Form, and Likert Muscle Soreness Scale) as well as psychological factors such as fear of movement, pain-related beliefs, and coping strategies (Tampa Scale for Kinesiophobia, Pain Beliefs Questionnaire, and Coping Strategies Questionnaire–Revised). This sample size provides 80% power at 5% significance to detect a medium effect size across 23 predictor variables, with Bonferroni correction and 10% loss-to-follow-up allowance. Data will be analyzed using 2-level growth curve modeling to characterize interindividual differences in motor behavior, cortical dynamics, and pain processing trajectories.

**Results:**

All study procedures have been approved by the Western University Health Science Research Ethics Board (review reference 2025-125757-103291). Funding was provided by the Canadian Institutes of Health Research under grant number 517783 for the period 2024‐2029. Recruitment for the study began in April 2025, and all data collection is expected to be completed by 2028. As of April 2026, we have enrolled 26 participants. Results are expected to be published at the end of 2028.

**Conclusions:**

The findings will advance understanding of motor behavior in pain and lay the foundation for personalized therapies, moving beyond current generic treatments that offer limited benefits.

## Introduction

Chronic pain affects at least 30% of people worldwide [1] and represents a growing public health crisis. Despite its substantial impact, effective treatment remains limited, in part because most interventions rely on standardized, symptom-based approaches that fail to address the diverse mechanisms driving pain across individuals. To advance pain care, a precision approach is needed, one that targets the right mechanism, at the right time, and in the right person.

One mechanism through which pain impacts individuals is motor behavior, a dynamic and adaptive system aimed at minimizing further harm and tightly linked to both the experience and persistence of pain [2]. In the short term, protective strategies, such as reducing movement amplitude or speed, help avoid pain provocation, prevent injury, and promote healing [3]. However, when maintained beyond the acute phase, these behaviors may become maladaptive, leading to stereotyped movement patterns, reduced muscle recruitment variability, increased cocontraction, altered tissue loading, and diminished proprioceptive feedback [2,4-7]. Over time, these changes are thought to produce mechanical, physiological, and neurological consequences that sustain pain. While the link between maladaptive motor behavior and persistent pain is widely acknowledged, empirical data on the underlying mechanisms remain limited. Cortical mechanisms may provide a key link between motor adaptation and persistent pain. Indeed, pain-related changes in corticomotor function can limit adaptive control of movement, favoring protective but inefficient strategies that may contribute to pain persistence [8].

Emerging evidence suggests that cortical mechanisms, particularly changes in sensorimotor cortex excitability and integration, may underpin the relationship between motor adaptation and pain [9-11]. On one hand, in the acute phase, reduced corticomotor excitability appears to serve as an adaptive strategy to protect the painful area and facilitate recovery [9]. However, if these reductions persist, they may impair motor learning and promote rigid, maladaptive movement patterns [12-14], increasing the risk of chronic pain [15,16]. Preliminary experimental data support this dual role [17], highlighting the need for further research to clarify how cortical dynamics interact with motor behavior across the transition from acute to chronic pain. On the other hand, sensorimotor integration, the process by which the central nervous system transforms sensory inputs into motor commands, is disrupted in persistent pain and may contribute to pain amplification [18,19]. While impairments in sensorimotor integration have been demonstrated in clinical pain conditions, evidence linking these changes to motor behavior remains limited [20,21]. Previous work showed that reduced sensorimotor integration is associated with decreased corticomotor output following a noxious stimulus [22]. However, how these alterations influence motor behavior over time, both within and between individuals, and how they interact with other cortical mechanisms, is still poorly understood.

While recent theories propose that cortical changes drive motor adaptations in pain, evidence remains mostly cross-sectional and focused on isolated mechanisms [2,5]. Progress is limited by the lack of human models inducing sustained, functionally relevant muscle pain, restricting the ability to track cortical and behavioral changes over time. This gap hampers our understanding of how pain-related motor adaptations develop and resolve, underscoring the need for longitudinal research to guide personalized pain management.

Here, we outline the experimental protocol and statistical analysis plan to examine individual differences in motor behavior (multidimensional movement task and muscular synergies), cortical mechanisms (corticomotor excitability, peak alpha frequency (PAF), and intracortical inhibition and facilitation), sensorimotor integration (sensory-evoked potentials [SEPs]), cognitive function (multisource interference task [MSIT] and questionnaires), and pain modulation (conditioned pain modulation [CPM]) in response to the progressive development, persistence, and resolution of clinically relevant elbow pain (intramuscular injection of nerve growth factor [NGF] into the extensor carpi radialis brevis [ECRB] muscle). We hypothesize that individuals with low sensorimotor cortex excitability, higher intracortical inhibition, reduced sensorimotor integration, central sensitization, and a passive cognitive response strategy will have less variable motor behaviors and more severe, longer-lasting pain.

## Methods

### Study Design

**Figure 1.**
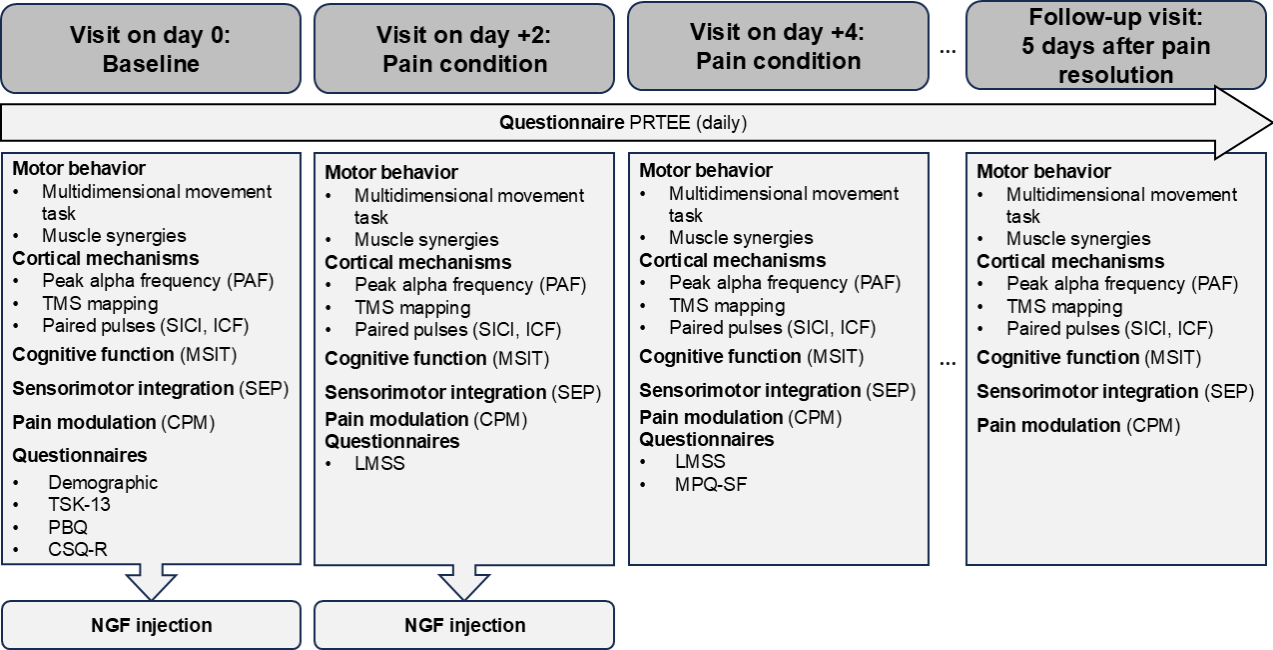
Study timeline. CPM, conditioned pain modulationConditioned Pain Modulation; PRTEE, Patient Rated Tennis Elbow Evaluation; TSK-13, Tampa Scale for Kinesiophobia - ––13 items; PBQ, Pain Beliefs Questionnaire; CSQ-R, Coping Strategies Questionnaire – –Revised; ICF: intracortical facilitation; LMSS, Likert Muscle Soreness Scale; MPQ-SF, McGill Pain Questionnaire - –Short Form; NGF, nerve growth factor; MSIT, Multisource Interference Task; NGF, nerve growth factor; PAF: peak alpha frequency; PBQ, Pain Beliefs Questionnaire; PRTEE, Patient-Rated Tennis Elbow Evaluation; SEP, sensory -evoked potentialsSensory Evoked Potentials; SICI: short-interval intracortical inhibition; TMS, Transcranial Magnetic Stimulation; TSK-13, Tampa Scale for Kinesiophobia–13 items.

A prospective longitudinal experimental study will be conducted to follow healthy individuals from the first induction of pain (day 0), as it develops and is sustained (day +2 and day +4), until 5 days after complete resolution of pain (Figure 1). Ethical approval has been obtained from the University of Western Ontario (reference 2025-125757-103291, January 9, 2025).

### Participants and Inclusion and Exclusion Criteria

A total of 150 healthy, pain-free individuals aged 18-65 years will be included in the study. Each participant will be asked to refrain from consumption of tobacco [23], caffeine [24], and recreational substances at least 2 hours before data collection [25], and short-acting analgesics (eg, acetaminophen) at least 6 hours before data collection [26], so as not to interfere with the perception of pain. Individuals presenting with a history of other major disorders, psychiatric conditions, or contraindications to the use of transcranial magnetic stimulation (TMS) will be excluded. Only right-handed people will be included due to the device used to analyze motor variability, which is custom built for right-handed individuals, and the presence of differences in sensorimotor integration [27] that would influence consistency and increase variability. Participants will be recruited from the community using newspaper or online advertisements and social media sites such as Facebook. Testing will take place at the Western Interdisciplinary Research Building on the University of Western Ontario campus at London (Ontario, Canada). Participants shall receive financial compensation for their participation.

### Data Collection Procedures

#### Primary Outcomes: Motor Behavior

##### Overview

**Figure 2.**
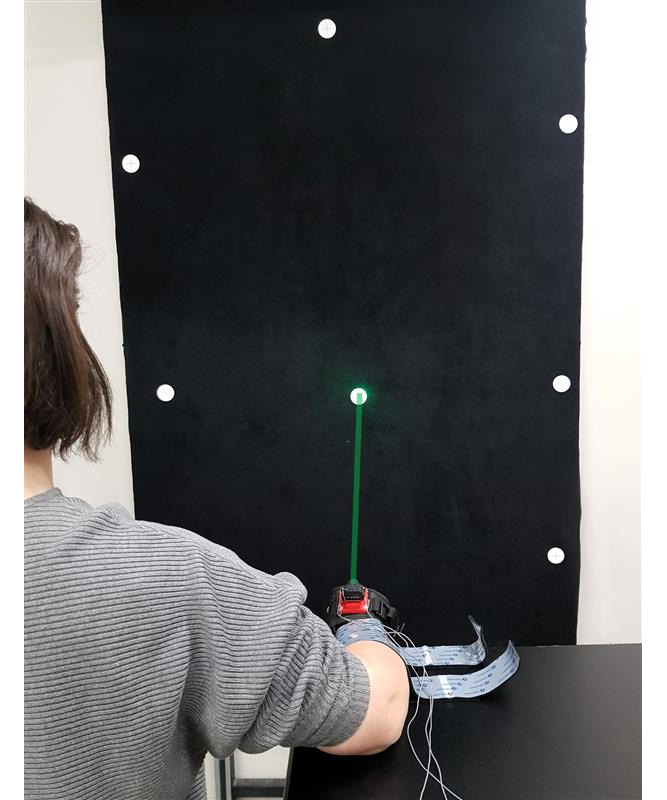
Experimental setup for the multidimensional wrist movement task. A laser pointer mounted on the dorsal surface of the glove will be used to reach 1 central and 8 peripheral targets, while the forearm will be secured to restrict movement to the wrist.

Although a predefined preprocessing pipeline will be applied, data-driven adjustments may be performed when warranted by data quality, statistical assumptions, or artifact structure. Every adjustment will be reported in the final study. Multidimensional movement tasks will be performed to assess both kinematic and muscle synergy variability. Participants will be seated on an adjustable seat with their right forearm resting on a table in neutral pronation-supination with approximately 70° of shoulder abduction. Their wrist will be positioned at the edge of the table with their hand extending beyond it, allowing the forearm to be supported without hindering wrist mobility. The task involves executing multidimensional wrist movements toward 9 white round targets (4.5 cm in diameter), 1 central and 8 peripheral, arranged in a circular configuration, separated by 45° from the central target, at a 1-meter distance on a black fabric wall. The 8 peripheral targets will be separated from the central one according to the functional ranges of motion of the wrist joint [28-31]. The targets will be arranged at 40° flexion (50% of the average amplitude of approximately 80°), or 84 cm below the central target; at 40° extension (57% of the average amplitude of approximately 70°), or 84 cm above the central target; at 15° radial inclination or abduction (75% of the average amplitude approximately 20°), or 27 cm to the left of the central target; and at 20° ulnar inclination or adduction (67% of the average amplitude approximately 30°), or 36 cm to the right of the central target. A green laser pointer, mounted on the dorsal surface of the right hand via an adjustable glove and aligned with the middle finger, will project a laser dot to indicate movement direction to participants. Participants will move the right wrist, keeping the elbow and forearm fixed by hook-and-loop fasteners on the table, to guide the laser pointer from the central target to each peripheral target and then returning to the central target (Figure 2). Movements will be performed in both clockwise and counterclockwise directions, alternating between the two. Each direction will be repeated 3 times, resulting in a total of 6 complete tours. Each tour consists of 16 movements, or 8 round trips from the central target to the 8 peripheral targets, with brief pauses between sequences. The task will follow a metronome cadence of 90 beats per minute, resulting in a total of 96 movements completed in 1 minute and 4 seconds, excluding pauses. Participants will be instructed to prioritize accuracy, aiming the laser pointer precisely at the targets and avoiding any corrective adjustments once a target has been reached. To establish a stable baseline for task performance, participants will undergo a separate familiarization time for all visits, just before the actual task. During this time, they will practice the task until they achieve a performance plateau, defined as a movement accuracy exceeding 95%. Previous research has demonstrated that 3 complete tours (or 48 trials) are sufficient to reach this plateau and that movement accuracy remains stable over several days [32]. Kinematic data will be recorded using a 3D Optotrak motion capture system (Northern Digital Inc) at a sampling frequency of 100 Hz tracking 3 infrared light-emitting diodes bound to the glove on the back of the right hand, roughly matching the distal end of the second and fifth metacarpal bones. Data will be processed by a customized Python (Python Software Foundation) script.

##### Kinematic Variability

Kinematic variability of the movement will be quantified each visit using the root-mean-square deviation (RMSD) of the 3D hand movement [33,34] around its mean trajectory. Hand position will first be defined across frames from raw position signals as the barycenter of all 3 active markers rigidly bound to the hand. Marker positions will, however, be low-pass filtered (zero-phase Butterworth filter, fourth order, cutoff frequency to be determined from the power spectrum density of actual data as described in Carbonneau et al [35]) before computing hand velocity as the symmetric derivative of their barycenter. The highest appropriate cutoff frequency across all participants will be applied to all so that no useful signal is lost and all participants’ data follow the same preprocessing. Filtering attenuates the noise amplification inherent to numerical differentiation, reducing spurious velocity spikes that could affect data segmentation. This approach avoids bias by ensuring that preprocessing decisions are not made separately for different visits or pain states and therefore cannot systematically favor one condition over another but could introduce some noise into a certain dataset.

Hand position data will first be roughly segmented into its 16 individual movements based on metronome beats. Finer movement onset and offset will then be defined as the time points at which the tangential velocity of the hand crosses a threshold of 10% of its peak value [36] and then remains beyond it (eg, sustained duration ≥50 ms), preventing transient velocity spikes to be misclassified as movement onset or offset. Trials in which the root-mean-square velocity during the presumed stationary phase exceeds a noise floor criterion (eg,>2% of peak velocity) will be automatically flagged as having irregular baseline activity for the eventual manual validation of the segmentation of all trials. This hierarchical, criterion-based approach minimizes segmentation errors that could artificially inflate RMSD estimates, while substantially reducing the proportion of trials requiring manual segmentation. Each kinematic segment will then be time-normalized to 100 data points.

For each visit, all 6 repetitions (3 clockwise turns and 3 counterclockwise turns) of the same movement will be averaged. The RMSD of concatenated individual hand trajectories relative to their own mean trajectory over the same visit will then be computed. This single scalar index of intravisit kinematic variability will then be entered into growth curve models to characterize longitudinal changes in movement dispersion across the 4 repeated visits independently of end point accuracy.

##### Muscle Synergies

Muscle synergies will be identified via electromyographic (EMG) recordings from 4 muscles involved in wrist flexion, extension, and radial or ulnar deviation, and selected for their accessibility to EMG recordings, superficial anatomical location, and distinct activation patterns during wrist movements. The *flexor carpi radialis* (FCR) was selected for its role in wrist flexion and radial deviation, with increased activation expected during supination and radial deviation. The FCR is located superficially on the anterior forearm, medial to the brachioradialis. Electrodes will be positioned one-third of the distance from the medial epicondyle of the elbow to the wrist crease, and just lateral (radial side) to the palmaris longus tendon (if present), with an interelectrode distance of 2 cm more distal [37]. The *flexor carpi ulnaris* (FCU), contributing to wrist flexion and ulnar deviation, will also be included. The FCU will be located along the ulnar border of the anterior forearm, adjacent to the ulna, with electrodes placed at one-third of the forearm length from the wrist, following the muscle’s longitudinal axis, with an interelectrode distance of 2 cm more distal [38]. For wrist extension, the ECRB will be targeted for its primary role in wrist extension and radial deviation. The ECRB will be located 1 cm lateral to a point that is 5 cm distal to the lateral epicondyle, along a line connecting the lateral epicondyle to the midline of the wrist, with an interelectrode distance of 2 cm more distal, to minimize muscle activity cross-contamination from adjacent muscles such as the extensor carpi radialis longus and the brachioradialis [39]. Finally, the *extensor carpi ulnaris* (ECU) will be selected for its contribution to wrist extension and ulnar deviation. Electrodes will be placed on the dorsal ulnar forearm, one-third of the way down the forearm from the lateral epicondyle (elbow) toward the wrist, on the ulnar (medial) side, and aligned with the muscle belly, with an interelectrode distance of 2 cm more distal [40,41]. A ground electrode will be placed over the olecranon. Every electrode will be placed on lightly abraded skin using Nuprep Skin Prep Gel (Weaver and Company) and alcohol. Each electrode will be placed according to the guidelines established by Hermens et al [42].

Every EMG signal will be collected using 24-mm diameter adhesive clipped disposable wired Ag/AgCl surface electrodes (Kendall H124SG), amplified (×1000), band-pass filtered (20‐1000 Hz) using a NeuroLog module NL125/NL126 (Digitimer), sampled at a rate of 5 kHz with a CED Micro 1401 mk II acquisition system, and recorded in CED Signal v6.0 software (Cambridge Electronic Design Limited).

EMG signals from the FCR, FCU, ECRB, and ECU muscles will be preprocessed using a customized MATLAB algorithm. The linear trend will be removed before filtering each signal with a fourth-order zero-lag band-pass Butterworth filter set between 20 Hz and 1000 Hz to remove motion artifacts at low frequencies and high-frequency noise. Full-wave rectification and smoothing through a fourth-order zero-lag low-pass Butterworth filter (cutoff frequency 5 Hz) will then yield EMG envelopes. Each muscle EMG envelope will subsequently be normalized by its peak activation observed across the whole visit, ensuring that synergy extraction reflects relative muscle coordination patterns independently of absolute activation magnitude. This normalization minimizes the influence of between-visit amplitude variability due to electrode placement, skin impedance, and amplifier gain, which are known to produce substantial day-to-day variability and can confound interpretation of activation amplitude [43-45]. This type of within-session peak normalization is commonly used when the focus is on coordination patterns and synergy structure, rather than on absolute activation levels [46,47]. Envelopes will then be cut using segmentation time stamps from kinematic data, and each of the 16 individual movements will be time-normalized to *t*=3000 samples. Individual movements will then be concatenated to represent the whole visit (6 turns) as a single data matrix. Spatial muscle synergies will be assessed each visit (*v*=4) from concatenated EMG envelopes by the nonnegative matrix factorization (NNMF) approach [48], as implemented in MATLAB (alternating least squares algorithm). The model will thus include for each participant and each visit the *m*=4 muscles (FCR, FCU, ECRB, and ECU) and all repeated (*r*=6) round trips corresponding to 16 pointing tasks (*k*=16). The NNMF approach can extract muscle synergies, *s* ranging from 1 to *m*–1=3. The spatial synergy (*U*, with *m* rows and *s* columns) and temporal coefficients (*C*, with *s* rows and *k × r × t* columns) will be identified from the preprocessed EMG matrix *M* (equation 1). This optimization will run 20 times to ensure the lowest possible reconstruction error (ε, with *m* rows and *k× r × t* columns; equation 1) since the NNMF method iterates from a random seed.


(1) M=U⋅C+ε

The reconstructed EMG matrix (equation 2) will be used to assess reconstruction quality using the variance accounted for (VAF) (equation 3). Unlike a coefficient of determination, the total sum of squares is not centered. VAF will be computed both globally and for each individual muscle (by setting the chosen muscle index and removing the summations over muscles in equation 3). A total of 20 VAF values will thus be associated with each participant [(*m* + 1) × *v*]. The minimal number of synergies required to reach satisfactory reconstruction quality will be optimized individually for each visit according to a threshold of 0.8 for each of the 5 associated VAF values (*m* individual muscle values plus global VAF).


(2) $$\hat{M}=U\cdot C$$

(3) $$VAF=1-\frac{\sum_{muscle=1}^{2}\sum_{time=1}^{t}{\left(M_{muscle,time}-{\hat{M}}_{muscle,time}\right)}^{2}}{\sum_{muscle=1}^{2}\sum_{time=1}^{t}M_{muscle,time}^{2}}$$

Synergy variability will involve only synergy activation variability since no motor adaptation should occur within 1 visit (no muscle synergy composition variability). The variability of activation coefficients will be quantified by the Pearson correlation coefficient (*r*), the maximum value of the normalized cross-correlation coefficient (*r*_max_), and the time shift between activation curves (lag_%_, expressed as a percentage of movement duration) between turns. All 3 variability metrics for each muscle synergy will be averaged across all pairwise combinations of the same 16 individual movements within a visit. *r, r*_max_, and lag_%_ values will finally be averaged over muscle synergies to reflect overall synergy activation variability. High muscle activation variability is reflected by low *r* and *r*_max_ values, as well as larger time shifts lag_%_. This approach is inspired by work based on the similarity of muscle synergy [49], although referring to activation similarity instead of activation variability. Metrics on intravisit muscle synergy activation variability (*r, r*_max_*,* lag_%_) will be compared between pain-free and pain-induced conditions regardless of muscle synergy composition between pain conditions.

### Cortical Mechanisms

#### Peak Alpha Frequency

Electroencephalographic (EEG) sensorimotor PAF will be recorded while the participants are comfortably seated. Scalp EEG will be recorded using the Brain Products platform (BrainVision Recorder, v1.22.0101 with actiCHamp Plus, Brain Products GmbH) at a sampling rate of 5000 Hz. Signals will be recorded from 63 active electrodes (actiCAP slim, Brain Products GmbH), embedded in an elastic cap (EASYCAP, EASYCAP GmbH) in line with the 10‐10 system [50]. Recordings will be referenced online to “FCz” with the ground electrode placed on “FPz.” Electrode impedances will be maintained below 5 kΩ during setup [51]. Once setup is complete, the lights will be switched off with ambient noise reduced to a minimum. Participants will be instructed to relax their muscles and keep their eyes closed while remaining awake. The resting-state EEG signal will then be recorded for 5 minutes.

EEG data processing will be conducted using custom MATLAB scripts implementing the EEGLAB (eeglab2025.0.0) and FieldTrip (fieldtrip-20251201) toolboxes [52]. The preprocessing pipeline will consist of data downsampled to 500 Hz, rereferenced from the online FCz reference to common average, band-pass filtered between 2 and 100 Hz with a notch filter at 60 Hz using a finite impulse response filter, and removal of auxiliary channels (galvanic skin response, heart rate, and respiration). A 5-minute resting-state window will be extracted. Channel data will be visually inspected in both time and frequency domains using spectral plots, and overtly noisy channels will be removed before rereferencing. Data will be segmented into 5-second epochs with no overlap (60 epochs total), and epochs containing marked artifacts will be manually rejected using FieldTrip’s visual rejection tool (±100 µV amplitude threshold). Independent component analysis will be applied using the runica method with 20 principal components retained. Components will be visually inspected via topographic maps, time-course plots, and frequency spectra to identify components with a clear alpha peak (8‐12 Hz) and scalp topography suggestive of sensorimotor cortex sources (bilateral distribution over central regions). Missing channels will be interpolated using the spline method with neighboring electrode templates prior to frequency decomposition. Mastoid electrodes (M1/M2) will be excluded from interpolation and final analysis. The power spectral density of the chosen sensorimotor component will be derived in 0.2 Hz bins across the 2‐50 Hz range using multitaper Fast Fourier Transform with a Hanning taper to reduce edge artifacts using the formula (equation 4). PAF will be calculated as the center of gravity (power-weighted mean frequency) within the 8‐12 Hz window, with equation 5.


(4) PAF=∑(f×P(f))/∑(P(f))

(5) $$CoG=\frac{\sum_{i=1}^{n}f_{i}\;a_{i}}{\sum_{i=1}^{n}a_{i}}$$

#### TMS Corticomotor Excitability

##### Overview

Motor-evoked potentials (MEPs) will be recorded from the right ECRB using wired Ag/AgCl clipped disposable adhesive 24-mm diameter surface electrodes (Kendall H124SG) and a TMS device (BiStim² and 200, Magstim Company Ltd) equipped with a figure-of-eight coil (P/N9925-00 S/N2349) outer diameter 90 mm. The signals will be amplified (×1000) and band-pass filtered (20‐1000 Hz) using a NeuroLog module NL125/NL126 (Digitimer), sampled at a rate of 5 kHz with a CED Micro 1401 mk II acquisition system and recorded in CED Signal v6.0 software (Cambridge Electronic Design Limited). The ECRB is a forearm muscle responsible for wrist extension and abduction, originating from the lateral epicondyle of the humerus and inserting at the base of the third metacarpal bone. Surface EMG electrodes will be positioned 1 cm lateral to a point that is 5 cm distal to the lateral epicondyle, along a line connecting the lateral epicondyle to the midline of the wrist, with an interelectrode distance of approximately 20 mm more distal, to minimize cross-contamination from adjacent muscle activity such as from the extensor carpi radialis longus and the brachioradialis [42,53,54]. The ground electrode will be placed on the olecranon to ensure minimal interference with the EMG signals. Before each application of EMG electrode, the skin will be lightly abraded using *Nuprep* skin prep gel (Weaver and Company) and then cleaned with alcohol.

To locate the optimal spot (hotspot) for the right ECRB muscle, the participant will be positioned comfortably with their forearm supported and relaxed. The coil will be used to initiate stimulation over the left M1, approximately 5 cm lateral from the vertex (Cz). To localize the Cz, the nasion-inion and interaural distances will be measured along the midline using a flexible measuring tape. The midpoint of each measurement will be marked, and their intersection will define Cz [55]. The coil will be systematically moved in 1-cm increments around this area, delivering 2‐3 stimuli at each location. The coil position that consistently elicits the largest MEPs, at the lowest stimulator intensity, in the ECRB will be identified as the hotspot. This location will be marked using a neuronavigation display system (Brainsight, Rogue Research Inc) to ensure reproducibility. Once the hotspot is determined and recorded in the neuronavigation system, the resting motor threshold (rMT) will be established using Motor Threshold Assessment Tool 2.0 software. Motor Threshold Assessment Tool 2.0 will use an automated procedure using a maximum likelihood parametric estimation by sequential testing algorithm [56] and will adjust stimulation intensities and prompt for input on whether an MEP ≥50 μV was observed after each pulse [57]. The final rMT will be defined as the lowest stimulation intensity that reliably elicits MEPs ≥50 μV in at least 50% of trials with 95% confidence.

##### Mapping

TMS mapping will be used to identify the motor cortical representation of the ECRB and assess corticomotor excitability, following a rapid and reliable procedure [58]. Rapid mapping will be performed over a 70 mm × 70 mm area flat grid within a neuronavigation display. The grid will be centered on the hotspot, extended +3.5 cm and −3.5 cm in the frontal (mediolateral) plane and +3.5 cm and −3.5 cm in the sagittal (anteroposterior) plane, and curved and superimposed on a scaled generic brain image and localized relative to Cz. The pseudorandom walk method will involve delivering 120 stimuli at 120% of day 0 rMT at randomized locations within the grid, with a minimum of 4-second interstimulus interval, ensuring an average density of 4 stimuli per cm² to achieve a dense distribution of stimulation sites for accurate interpolation and peak identification [59-61]. The neuronavigation display will be monitored to confirm even stimulus distribution and prevent consecutive stimulations from being too close [62].

Motor maps will be generated offline using a custom Python script (scipy.interpolate.griddata) with a triangular linear interpolation used to create a continuous surface map from 3D MNI stimulation coordinates and corresponding peak-to-peak MEP amplitudes, while trials with background EMG activity will be excluded. The resultant motor map will be divided into 2500 partitions, each assigned an interpolated MEP value based on the nearest acquired data. Map volume will be calculated following van de Ruit et al [62], although a 50-μV minimum threshold will be used instead of the “greater than 10% of the maximum MEP” threshold, to include more responses and minimize outlier influence [58].

##### Paired Pulse

Paired pulse TMS will be performed to assess the short-interval intracortical inhibition (SICI) and the intracortical facilitation (ICF) for the right ECRB muscle following a standardized protocol [63]. Each condition will be tested in 12 trials at interstimulus intervals of 2 ms for SICI and 13 ms for ICF. As previous studies have shown that muscle pain reduces MEP amplitude [11,64] and that the size of the test response influences SICI magnitude [65], a 0.3-0.4-mV test response will be selected to maintain consistency across visits, even if corticomotor excitability decreases due to pain. The conditioning stimulus will be set at 90% rMT. An additional 12 unconditioned test trials will be recorded, totaling 36 trials, which will be presented in pseudorandom order. MEP responses will be measured as peak-to-peak amplitudes, and conditioned responses will be expressed as a proportion of the unconditioned test response.

### Sensorimotor Integration

SEPs will be recorded following electrical stimulation of the radial nerve. The cathode (black negative electrode) will be positioned 1.5 cm radial to the dorsal tubercle of the radius, targeting the superficial branch of the radial nerve [66]. The anode (red positive electrode) will be placed over the tendinous area in the distal third of the forearm. Electrodes will be spaced 3 cm apart. The forearm will be supinated with slight wrist extension [67]. Two blocks of 500 stimuli of 1-ms square pulse duration at a frequency of 2 Hz will be delivered, and a randomized interstimulus interval of 20% (0.4‐0.6 seconds) will be programmed to reduce habituation. The perceptual threshold will be determined by starting with a low-intensity stimulus and gradually increasing it until the participant reports a sensation. The stimulation intensity for the experiment will be set just below pain threshold without creating muscle twitches. Two blocks of 500 stimuli will be delivered while ensuring that the participant remains relaxed and minimizes movement to reduce artifacts.

The same EEG setup and preparation will be used as for the PAF. Data from the 2 blocks of 500 stimuli will be analyzed to examine the N_20_-P_25_-N_33_ complex as a marker of early cortical processing of somatosensory input. Data will be processed using MATLAB, resampled to 500 Hz, and auxiliary physiological channels will be excluded. EEG will be rereferenced to the right mastoid electrodes (M2). Band-pass filtering will be applied from 0.5 to 40 Hz (zero-phase finite impulse response), followed by a 59‐61 Hz notch filter. Data will be epoched from −50‐+150-milliseconds around each stimulus onset and baseline-corrected using the prestimulus interval (−50‐0 ms). Artifact rejection will be proceeded in 2 stages: automated *z*-value thresholding (cutoff = 4, FieldTrip *ft_artifact_zvalue*), followed by optional interactive summary rejection (*ft_rejectvisual*). All rejected trials will be logged with their rejection stage.

SEP will be extracted at electrode C3, contralateral to the stimulated right wrist, as it overlies the primary somatosensory cortex generating the early cortical response [68]. The area for the N_20_–P_25_–N_33_ complex within a window of 18‐40 ms will be computed per trial [22].

### Cognitive Function and Related Brain Activity

It is hypothesized that motor behavior in response to pain is influenced by individual cognitive coping strategies toward threat. People typically adopt either a passive coping strategy, focusing on pain and avoiding movement, or an active coping strategy, focusing on task completion and problem-solving. Previous work has shown that during a cognitive task performed in pain, individuals naturally adopt one of these strategies, which impacts performance [69-73]. Passive coping is associated with worsening performance, while active coping improves it [71]. This project will be the first to explore how these cognitive strategies relate to motor behavior in pain.

MSIT is a cognitive task designed to probe conflict resolution and cognitive control by activating the anterior cingulate cortex and frontoparietal networks [74]. MSIT combined with EEG allows investigation of how cognitive load influences pain perception and how pain affects cognitive performance. The MSIT will be modified from the original version of the MSIT [75], will be run using PsychoPy (version 2025.2.1; Open Science Tools Ltd), and will include 2 levels of difficulty: “low load” and “high load” [76]. In both levels, participants will identify the number on the screen that differs from the other 2 characters. They will use their right index, middle, and ring fingers to press the corresponding buttons, labeled “1,” “2,” and “3” arranged from left to right on a separated 3-keys keyboard. The MSIT introduces cognitive conflict by manipulating spatial and size-related features. For the easy level, 2 of the 3 characters are identical, and the position of the target number aligns with its corresponding button on the response box (eg, “323” with the correct answer being “2”). For the difficult level, spatial and size conflicts are introduced, meaning the target number does not align spatially with its button (eg, “311” with the correct answer being “3”). Each stimulus will remain on the screen until the participant presses a button, after which there will be a 1.25-second delay before the next stimulus appears [77]. To minimize cognitive fatigue, the task will be divided into 4 blocks per difficulty level (4 low load +4 high load), with 30-second rest periods between blocks. The participant will first be offered a training session consisting of an easy block and then a difficult block (repeatable if the participant wishes), followed by 8 blocks of 24 random trials in the fixed order load low-high-low-high-low-high-low-high, each separated by 30-second rests. For visits on day +2 and day +4, a tight armband will be applied over the site of injection to generate pain approximately 3/10 if the pain is less than this value at rest. We will examine behavioral performance for the 2 levels of difficulty, focusing on accuracy, defined as the proportion of correct, and reaction time, defined as the elapsed time between stimulus onset and the participant’s motor response.

### Pain Modulation

Central sensitization, defined as an increased sensitivity of spinal and cortical neurons, and impaired descending pain modulation are key mechanisms influencing motor behavior in pain. These changes may interfere with the link between pain experience and motor behavior, as movement recovery may not reflect tissue healing [78]. Central sensitization can sustain pain beyond peripheral injury and interfere with the analgesic effects of movement. Moreover, it may compete with motor learning processes, potentially impairing motor adaptation [79]. While animal and human studies suggest a relationship between central sensitization and altered motor behavior, evidence remains limited [80-84].

CPM will be used to assess the inhibitory pain mechanisms, using 2 different types of test stimuli according to guidelines [85-87]. In our study, this assessment will involve measuring changes in both heat pain thresholds (HPTs) and pressure pain thresholds (PPTs) through 3 consecutive measurements, conducted twice and separated by a conditioned stimulus to activate endogenous pain modulation mechanisms [88].

The HPTs will be determined using a thermode (TCS-II, QST.Lab) equipped with a 4.5 cm² rectangular probe (T09). First, participants will undergo a familiarization phase with the thermode: holding the thermode in the palm of their hand and experiencing the temperature rise. Starting from a baseline temperature of 32 °C, thermal stimuli will be applied on the proximal third of the anterior surface of the right forearm of the participants with a gradual increase of 1 °C per second until the participant reports the first sensation of pain by pressing a response button. The temperature at this moment will be recorded as the HPTs. To ensure reliability, 3 consecutive measurements will be performed, and the mean value will be calculated and will be used for analysis. After each trial, the temperature will return to baseline in less than 1 second. Safety cutoff temperatures will be set at 50 °C to prevent tissue damage.

The PPTs will be assessed using a pressure algometer (Mxmoonfree ZMF-300N Portable Digital) equipped with a flat metallic round tip of 1.4 cm in diameter by exerting a constant pressure of 50 kPa per second (approximately 0.5 kg/cm^2^ s). During the assessment, the participants’ forearm will be placed on a table while an examiner will exert pressure perpendicular to the skin. Participants will be asked to say “stop” to signal when the sensation changes from pressure to pain. When the participant responded “stop,” the pressure will stop, the device will be removed from the skin, and the pressure value recorded. Three recordings will be made and averaged (1-minute intervals) over the right ECRB (on injection site) and used for analysis.

Both HPTs and PPTs will be assessed before and directly after a conditioning cold pressor test (CPT) in balanced order to allow us to measure several excitatory and inhibitory pain mechanisms. The CPT will be administered by immersing the participant’s left forearm in cold water at 10 °C with a water flow for 2 minutes [89].

The magnitude of CPM will be calculated by subtracting the average HPTs and PPTs measured after the CPT stimulus from those recorded before the CPT stimulus (pre-post). Therefore, a negative value will indicate pain inhibition (CPM response), while a positive value will suggest pain facilitation [90].

### NGF Injection

Repeated intramuscular injection of NGF induces progressive, persistent muscle pain that is mechanistically, functionally, and clinically relevant. Importantly, NGF induces a gradual onset of pain without tissue injury [91], mimicking key features of persistent musculoskeletal pain, including movement-evoked pain, spreading hyperalgesia, and functional limitation [92,93], which resolve spontaneously around 25 days after injections have ceased [39,94]. These characteristics make the NGF model a safe and clinically relevant approach to investigate the cortical mechanisms underlying pain-related motor behavior and its individual variability, as it replicates the time course, movement-evoked nature, and sensitization processes characteristic of persistent musculoskeletal pain.

After cleaning the skin with alcohol, a solution of recombinant human NGF (Active GMP Recombinant Human NGF Protein from Creative BioMart Inc) will be administered as a bolus injection of 5 μg, dissolved in 0.2 mL of sterile water, into the muscle belly of the right ECRB muscle at the end of visits on days 0 and day +2. The injection will be administered using a 1-mL syringe with a 27-gauge, 1-inch needle. The site of injection will be located 1 cm lateral to a point that is 5 cm distal to the lateral epicondyle, along a line connecting the lateral epicondyle to the midline of the wrist [39,95]. The participant’s upper limb will be supported on a table, with the elbow flexed to 90° and the forearm in pronation to ensure proper positioning and accessibility to the target area. A permanent marker will be used to circle the injection site to maintain consistent injection placement in the ECRB muscle throughout the visits. In order to ensure optimal effectiveness of the hyperalgesia, we will keep the NGF at a temperature of −80 °C until a few minutes before the injection [96].

### Questionnaires

All questionnaires will be computerized and sent via Qualtrics Survey Software (version XM; Qualtrics, LLC). Patient-Rated Tennis Elbow Evaluation (PRTEE) is a 15-item questionnaire designed to measure forearm pain and disability in patients with lateral epicondylitis, also known as tennis elbow [97-99]. It consists of 2 subscales: a 5-item pain subscale and a 10-item function subscale, with each item rated on a scale of 0-10. The pain subscale is scored out of 50, and the functional subscale is divided by 2 to bring it down to a score out of 50 for a total score out of 100. The PRTEE has demonstrated high test-retest reliability (intraclass correlation coefficient [ICC]=0.96), internal consistency (Cronbach α=0.96) [99], and shows good construct validity against other measures such as the Disabilities of the Arm, Shoulder and Hand (*r*=0.81) [100]. The PRTEE is considered a reliable and valid tool for assessing pain and functional disability in patients with lateral elbow tendinopathy.

Tampa Scale for Kinesiophobia (TSK) is a widely used self-report questionnaire using a 4-point Likert scale, designed to assess fear of movement [101]. The objective of the TSK is to quantify kinesiophobia, defined as an irrational and debilitating fear of physical movement resulting from a feeling of vulnerability to painful injury or reinjury [102]. The TSK has demonstrated good to excellent test-retest reliability (ICC=0.77-0.99) and good internal consistency (Cronbach α=0.68-0.91) across various versions [103,104]. We have selected the TSK-13 (13 items), which presents good construct validity and strong psychometric properties, for a reduced completion time [103,105]. Participants will be asked to rate their agreement with the item statements from “*strongly disagree*” to “*strongly agree*,” with total scores ranging from 13 to 52, where higher scores indicate greater kinesiophobia [106]. Because the questionnaire was initially designed for patients with musculoskeletal pain, the questionnaire’s instructions were adapted to align with our healthy participants, following the validated TSK-G version for the general population [107], as done in previous studies [108-110]. The revised instructions were as follows: “We are interested in the types of thoughts and feelings that you might have in response to pain. Please read each of the following statements and check the box that best represents your thoughts and feelings while experiencing muscle pain.”

Pain Beliefs Questionnaire (PBQ) is a self-report instrument designed to assess individuals’ beliefs about the causes and consequences of pain [111]. Initially developed as a 20-item questionnaire, it was later refined to a 12-item version comprising 2 subscales: the Organic Beliefs Scale (8 items) and the Psychological Beliefs Scale (4 items) [112]. The PBQ evaluates pain-related beliefs, particularly the perceived organic nature of pain and the role of psychological factors in pain perception. The questionnaire has demonstrated strong construct validity, with patients with chronic pain and healthy controls exhibiting significant differences in their endorsement of organic and psychological beliefs [111]. It also shows good internal consistency (Cronbach α=0.68-0.91) across different versions [113]. Respondents rate their agreement with each statement on a 6-point Likert scale from “*always*” to “*never*.” The PBQ has been effectively used in studies on chronic back pain and is applicable to both individuals experiencing pain and healthy controls [112]. Its ability to assess pain beliefs across diverse populations makes it a valuable tool for understanding pain perceptions and informing pain management strategies.

Coping Strategies Questionnaire–Revised (CSQ-R) is a 27-item self-report instrument designed to assess cognitive and behavioral strategies for coping with pain [114]. It evaluates 6 key domains: Distraction, Catastrophizing, Ignoring Pain Sensations, Distancing from Pain, Coping Self-Statements, and Praying. The CSQ-R demonstrates a robust factorial structure, high reliability, and strong validity across multiple studies. Its subscales show excellent internal consistency (Cronbach α=0.914-0.961) and good to excellent test-retest reliability (ICC=0.850-0.918). Construct validity is supported by significant correlations with measures of pain, disability, depression, and other coping inventories. Administered as a self-report measure, the CSQ-R requires respondents to rate the frequency of their use of each coping strategy on a Likert scale from 0 “*never*” to 6 “*always*.”

Likert Muscle Soreness Scale (LMSS) is a self-report tool designed to assess perceived muscle soreness, particularly in the context of delayed onset muscle soreness. Its primary objective is to quantify the severity of muscle soreness and its impact on functional tasks using a 7-point scale ranging from 0 (no pain) to 6 (severe pain limiting movement) [115]. It has demonstrated good construct validity, with significant correlations (*r*=0.65-0.941) to other pain measures, such as the visual analogue scale, and its reliability is sensitive to changes in soreness over time [116]. Administered as a self-report measure, participants rate their soreness based on predefined descriptors. The LMSS has been validated in various populations, including long-distance runners and soccer players, confirming its applicability across different athletic groups [115]. The LMSS will be tailored to best match the elbow pain we will induce with the NGF injection by adding the italicized words at the end of the instructions as follows: “Please tick the sentence below that best describes your level of muscle soreness over the past 12 hours *related to the injection*.” For the same reason, the last 4 items will be adapted for the elbow pain as follows: “A light muscle soreness when I move my elbow/wrist in a large amplitude or/and when I handle an object with force”, “A light muscle soreness, stiffness or weakness when I move my elbow/wrist or/and when I handle an object with finesse,” “A moderate muscle soreness, stiffness or weakness when I move my elbow/wrist or/and when I handle an object with finesse” and “A severe muscle soreness, stiffness or weakness that limits the ability to move my elbow/wrist or/and handle an object.”

McGill Pain Questionnaire–Short Form (MPQ-SF) is a widely used self-report instrument designed to assess the multidimensional nature of pain [117]. The MPQ-SF consists of 15 descriptors (11 sensory and 4 affective) each rated on a 0 “none” to 3 “severe” intensity scale. The MPQ-SF provides a concise yet comprehensive measure of both sensory and affective pain dimensions, making it suitable for clinical and research settings. The MPQ-SF has demonstrated strong psychometric properties across various chronic pain populations, with high internal consistency (Cronbach α=0.73-0.89) [118] and excellent test-retest reliability (ICC=0.96) [119]. Validity studies support its construct and concurrent validity, showing strong correlations with other pain measures and sensitivity to changes in pain intensity.

*Body diagram* in the McGill Pain Questionnaire is a widely used tool for assessing pain location and distribution. It provides a visual representation of a patient’s pain by allowing them to mark affected areas on anterior and posterior body schematics including 9 areas: trunk, head and neck, shoulder, arm, elbow, forearm, hand, thumb, and fingers. The test-retest for measuring pain distribution demonstrated a moderate to excellent reliability (ICC=0.58-0.94), but which shows a great variability of reliability to assess the localization of pain, according to the specific circumstances of the evaluation (κ=0.13-0.85) [120]. The administration of body diagrams is typically self-reported, with patients marking their pain on the diagram following standardized instructions.

### Sample Size

The trial is conservatively powered based on the use of separate multiple regression models to determine which mechanistic variables predict motor outcome at each time point. This calculation is based on 20 mechanistic variables: sensorimotor cortex excitability × 4 (map, SEPs, SICI, and ICF), sensorimotor integration × 1 (PAF-EEG), pain modulation × 2 (HPTs and PPTs), cognitive response × 4 (MSIT-easy-hard × 2 conditions), and physical activity or cognitive response × 6 (MPQ-SF, TSK-13, CSQ-R, PRTEE, LMSS, and PBQ), plus 3 a priori selected interactions and demographic variables × 3 (age, sex, and gender). We require 150 participants to achieve a power of 80% with a 5% significance level to detect a medium effect size and allowing for 10% loss to follow-up (based on our prior studies). The significance level was adjusted for multiple testing using a Bonferroni correction with an assumed correlation between predictors of 0.1. Using an alternative approach of 5‐10 participants per parameter, a sample of 150 could accommodate 15-30 parameters.

### Statistical Analysis

The analysis will be performed using growth curve modeling (GCMs) with the R software (R Core Team; R Foundation for Statistical Computing) for the 2 main outcomes [121]. Advantages to the use of GCM to examine our aim are as follows: (1) GCM enables consideration of the time-varying nature of both outcome (motor behavior) and the mechanistic (central nervous system) variables in response to pain; (2) GCM allows mechanistic variables to be simultaneously adjusted, permitting estimation of the unique contribution of each mechanism to motor behavior, as well as interaction between mechanisms; and (3) GCM can use unevenly spaced measurements and all available data, even in the presence of missing data at random. Thus, we will investigate whether mechanistic variables (and the interaction between them) at each time point, and the change between time points, predict motor behavior. To address individual variation in motor behavior, the model will use a 2-level nested structure. Level 1 will be the within-participant model and level 2 the between-participants model. The within-participant component allows each participant to have a unique motor behavior via random effects, and the between-participants component represents variation in motor behavior between individuals with similar mechanistic characteristics. The issue of controlling many covariates in statistical modeling will be addressed using modern adjustment techniques (eg, shrinkage estimation or exposure modeling) [122]. In addition, bootstrapping will be used to identify variables to be included in the final model [123]. Two models will be developed, 1 for kinematic movement variability and 1 for muscle synergies.

### Ethical Considerations

All procedures will be conducted in accordance with the Declaration of Helsinki. Written informed consent will be obtained, and participants will be free to withdraw from the study at any time. Patient data will be stored in a secure and structured database requiring access authorization via key card. The study data from the questionnaires will be collected via Qualtrics, a secure, web-based software platform designed to facilitate data collection and management for research purposes. Data are available from the corresponding author after agreement of the ethics committee. All procedures will be conducted under continuous supervision by trained personnel, with safety monitoring implemented throughout NGF-induced pain and neurophysiological testing. Participants will undergo comprehensive screening prior to inclusion, including TMS-specific safety questionnaires and exclusion of individuals with contraindications (eg, neurological, psychiatric, or implanted electronic or metal devices). During experimental sessions, participants will be closely monitored for adverse events (eg, excessive pain, dizziness, anxiety, and skin irritation), and all procedures (TMS, EMG, EEG stimulation, pressure, and thermal testing) can be immediately paused or terminated upon request. NGF injections (GMP grade), which have been documented as safe in humans [124-126], will be administered using sterile techniques, and expected transient muscle soreness will be monitored during visits; participants will be instructed to report any unexpected or prolonged symptoms. Standardized adverse event reporting procedures will be followed, including documentation, clinical assessment, and, if necessary, referral for medical evaluation.

## Results

### Overview

All study procedures have been approved by the Western University Health Science Research Ethics Board (review reference 2025-125757-103291). Funding was provided by the Canadian Institutes of Health Research under grant number 517,783 for the period 2024‐2029. Recruitment for the study began in April 2025, and all data collection is expected to be completed by 2028. As of April 2026, we have enrolled 26 participants. To date, 4 participants have been excluded: 1 due to a lack of pain, 1 due to a poor-quality EEG signal, and 2 due to the impossibility of obtaining TMS hot spot. Results are expected to be published at the end of 2028. Data will be made available on request. The results will be disseminated through peer-reviewed publications and presentations at national and international conferences.

### Data Quality Control and Handling of Missing Data

Participants who do not develop NGF-induced pain (PRTEE=0 on days +2 and +4) will be excluded. Data quality criteria will be applied across modalities, including successful elicitation of MEPs for ≥90% of TMS pulses and ≥90% marker tracking for motion capture. EEG analyses will be restricted to high-quality, artifact-free data: segments contaminated by eye movements, muscle activity, or excessive amplitudes (±75‐100 µV) will be rejected using automated or visual procedures. For resting-state analyses, PAF will be estimated only when at least approximately 3 minutes of clean data are available and a clear, stable alpha peak (8‐12 Hz) is identifiable; datasets with excessive channel noise (>10% bad channels), insufficient data, or unstable spectra will be excluded. For somatosensory-evoked potentials, trials with artifacts will be discarded, and analyses will require ≥80% of clean trials; averaged waveforms must show identifiable and temporally consistent components (eg, N20 and P25); otherwise, they will be excluded [127-129]. Missing data across visits and modalities will be handled using full information maximum likelihood within growth curve models under a missing-at-random assumption, and all exclusions and missing data patterns will be reported.

## Discussion

### Principal Findings

This study is expected to show that NGF-induced sustained muscle pain alters motor coordination patterns, characterized by reduced kinematic variability and increased muscle synergy activation consistency. These changes are hypothesized to emerge during pain development (days +2 to +4) and to persist beyond pain resolution. We hypothesize that individuals with lower sensorimotor cortex excitability (reduced TMS map volume), elevated intracortical inhibition (increased SICI), impaired sensorimotor integration (reduced SEP area), and passive cognitive coping strategies will exhibit the most maladaptive motor responses, manifesting as stereotyped, low-variability movement patterns.

### Comparison With Prior Work

While acute pain studies document immediate motor inhibition and reduced corticomotor excitability [11,130], our longitudinal NGF model extends this work by capturing the transition from acute protection to potential maladaptation across pain development and resolution [131]. Unlike cross-sectional clinical pain research showing reduced movement variability in chronic conditions [132,133], our experimental pain model will allow us to examine the temporal precedence of cortical changes over motor adaptations. Preliminary NGF work demonstrates pain hypersensitivity and altered PPTs [131,134], but our comprehensive assessment linking cortical excitability, sensorimotor integration, cognitive coping, and multidimensional motor outcomes represents a novel mechanistic synthesis. The hypothesis that passive cognitive strategies predict maladaptive motor responses builds on evidence that such strategies impair cognitive performance during pain [135-137], suggesting a potential extension of this relationship to the emergence of rigid movement patterns.

### Strengths and Limitations

Strengths include the longitudinal design tracking pain transition across multiple time points, comprehensive multimethod assessment (TMS/EEG/EMG/kinematics and questionnaires), and GCM accommodating individual differences and missing data. The NGF model produces clinically relevant sustained forearm pain-mirroring lateral epicondylalgia, enhancing translational validity. Limitations include reliance on healthy participants, limiting direct generalizability to clinical pain populations despite mechanistic parallels.

### Future Directions

Future research should validate these cortical-motor biomarkers in chronic pain populations to establish clinical usefulness. Personalized neuromodulation targeting identified cortical mechanisms (eg, inhibitory rTMS for elevated SICI) warrants investigation. Integration with structural or functional neuroimaging and machine learning trajectory classification could refine precision medicine approaches. Finally, active coping interventions during pain development may prevent maladaptive motor learning, warranting randomized trials.

### Dissemination Plan

Findings will be disseminated via open-access publication in high-impact pain journals, preregistration updates on Open Science Framework, conference presentations (eg, IASP 2028, Society for Neuroscience), and community seminars. Raw data, analysis scripts, and processing pipelines will be deposited in Open Science Framework post publication.

### Conclusions

This protocol describes a longitudinal experimental study designed to examine how NGF-induced sustained muscle pain influences motor adaptation, cortical excitability, sensorimotor integration, and pain-related cognition in healthy participants. By combining repeated assessments across the transition from pain development to recovery, the study will help clarify which neurophysiological and behavioral factors are associated with maladaptive movement responses and which may predict greater persistence of altered motor control.

The findings are expected to improve understanding of the mechanisms linking pain and movement and to provide a framework for future work in chronic musculoskeletal pain. In doing so, this study may support the development of more targeted, mechanism-based interventions for pain-related motor dysfunction.

## Supplementary material

10.2196/99833 Peer Review Report 1 Peer review report by the Canadian Institutes of Health Research.

## Abbreviations

CPM: conditioned pain modulation
CPT: cold pressor test
CSQ-R: Coping Strategies Questionnaire–Revised
ECRB: extensor carpi radialis brevis
ECU: extensor carpi ulnaris
EEG: electroencephalography
EMG: electromyographic
FCR: flexor carpi radialis
FCU: flexor carpi ulnaris
GCM: growth curve modeling
HPT: heat pain threshold
ICC: intraclass correlation coefficient
ICF: intracortical facilitation
LMSS: Likert Muscle Soreness Scale
MEP: motor-evoked potential
MPQ-SF: McGill Pain Questionnaire–Short Form
MSIT: multisource interference task
NGF: nerve growth factor
NNMF: nonnegative matrix factorization
PAF: peak alpha frequency
PBQ: Pain Beliefs Questionnaire
PPT: pressure pain threshold
PRTEE: Patient-Rated Tennis Elbow Evaluation
RMSD: root-mean-square deviation
rMT: resting motor threshold
SEP: sensory-evoked potential
SICI: short-interval intracortical inhibition
TMS: transcranial magnetic stimulation
TSK: Tampa Scale for Kinesiophobia
VAF: variance accounted for
