# Supplementary material for: Understanding Motor Adaptation in the Transition to Sustained Pain: Protocol for a Longitudinal Experimental Study
Source: JMIR Res Protoc. 2026 Jun 18;15:e99833. doi: 10.2196/99833 (PMC13277826; doi:10.2196/99833)
Supplement: Peer Review Report 1 [file resprot-v15-e99833-s001.pdf]

**Review Type / Type d'évaluation:**	Reviewer 1 / Évaluateur 1
**Name of Applicant / Nom du chercheur:**	Schabrun, Siobhan
**Application No. / Numéro de demande:**	517783
**Agency / Agence:**	CIHR/IRSC
**Competition / Concours:**	Project Grant/Subvention Projet
**Committee / Comité:**	Movement & Exercise/Mouvement et exercice
**Title / Titre:**	Unravelling the mechanistic architecture of motor behaviour in pain

Adjudication Criteria/Critères de sélection

Initial Score/Cote Initiale: 4.9

Top/Bottom Selection/Groupe supérieur/inférieur

- (●) **Top/Groupe supérieur**
- ( ) **Bottom/Groupe inférieur**

**Review Type / Type d'évaluation:**	Reviewer 1 / Évaluateur 1
**Name of Applicant / Nom du chercheur:**	Schabrun, Siobhan
**Application No. / Numéro de demande:**	517783
**Agency / Agence:**	CIHR/IRSC
**Competition / Concours:**	Project Grant/Subvention Projet
**Committee / Comité:**	Movement & Exercise/Mouvement et exercice
**Title / Titre:**	Unravelling the mechanistic architecture of motor behaviour in pain

Summary of Application/Résumé de la demande:

The PI is a mid-career researcher in PT and neuroscience who joined UWO in 2022 (previous work in Australia). Very successful record of funding (incl NSERC and significant NIH award) and outstanding publication record. Research on mechanisms, treatment, and prevention of chronic pain, focusing on understanding why some people develop chronic pain while others do not; identifying cortical biomarkers for pain prognosis; and using non-invasive brain stimulation intervention for treatment/prevention of pain. Of particular relevance to current proposal is their recent finding that individuals with lower sensorimotor cortical excitability during the acute stage of low back pain were more likely to experience pain at 6-months follow-up, and theory is that the lower cortical excitability during acute pain may be an adaptive strategy that restricts movement, and therefore protective from further injury. However, they surmise that this initial lower excitability may not be beneficial in long run because it limits motor variability and this less variable, more stereotypical movement pattern may reinforce maladaptive patterns, perpetuating cycle of pain.

Another important contribution to pain field is that they have developed a human pain model to represent the transition from acute to chronic pain, allowing them to track cortical adaptation and motor behaviour over time as pain develops, persists, and then resolves. They do this by intramuscular injection of NGF to mimic the clinical situation. Pain develops slowly following NGF injection – they have shown in previous publication that NGF mimics pain associated with slowly-developing muscle soreness that is related to movement, and that it resolves around 25 days after the injections.

This will be longitudinal study, plan to enrol 220 healthy, pain-free adults (50:50 M:F), all will receive intramuscular NGF injection in ECRB (mimicking tennis elbow), and then undergo repeated assessments at baseline, then days 2, 4, 8, 21, and 40.

Primary outcome is a motor task that requires participants to move their wrist in order to point at projected targets arranged in circular pattern. Measures will be EMG variability and kinematic variability across the movement cycle.

Also will use neurophysiology for battery of assessments to track changes in sensorimotor cortical excitability, intracortical inhibition and facilitation, central sensitization and cognitive function (e.g. cognitive set, coping strategy, and PA). Data will be analyzed using growth curve modelling, and separate models (one for EMG variability, the other for kinematic variability) will be generated.

Strengths and Weaknesses/Forces et faiblesses:

Strengths:

Convincing human model mimicking clinical experience of intramuscular pain that allows for study of the change in cortical activity over time, associated with the development, persistence, and ultimately resolution of pain.

Solid theoretical basis, hypothesis-based approach to understanding the mechanisms that could potentially explain why some people develop chronic pain and others do not.

Well-designed study with large, but feasible, sample size that will allow for large-scale data to examine cortical mechanisms and motor behaviour during the pain development.

Excellent and convincing preliminary data showing subjective experience of pain induced by NGF injection (pain rating scale and pressure pain threshold); these data also point to potential M:F differences. Also preliminary data supportive of one part of hypothesis that acute pain is linked to GABA-ergic activity (using TMS-EEG), as well as hypothesis that cortical excitability in acute stage of pain could predict who experienced recovery (and who did not) of pain at 6 months. Finally, the NGF model yields range of variability across subjects in their response over time, supporting this as a model that could mimic the clinical situation and therefore provide an avenue for understand why some people recover and others do not.

Strong team, each member brings requisite expertise to complement the PI, including new addition of biostatistician to oversee the GCM.

Identify viable next steps in research on interventions that could be translated from these findings, i.e. if confirm low cortical excitability associated with maladaptive motor behaviour and therefore risk of persistent pain, could test the effect of interventions (e.g. brain stim) to raise excitability during acute phase of pain.

Previous concerns were addressed, and help to strengthen the proposal (e.g. inclusion of additional Day 40 to confirm return to baseline after NGF injection).

Weaknesses:

There remains the question of the extent to which the NGF injection model reflects the clinical scenario leading to chronic pain, but this consideration is recognized by the applicants, and there is multiple converging evidence supporting the validity of the NGF model to model the development of chronic pain. This work will be important to lay the foundation for continued work to develop the model.

**Review Type / Type d'évaluation:**	Reviewer 1 / Évaluateur 1
**Name of Applicant / Nom du chercheur:**	Schabrun, Siobhan
**Application No. / Numéro de demande:**	517783
**Agency / Agence:**	CIHR/IRSC
**Competition / Concours:**	Project Grant/Subvention Projet
**Committee / Comité:**	Movement & Exercise/Mouvement et exercice
**Title / Titre:**	Unravelling the mechanistic architecture of motor behaviour in pain

Budget Recommendation/Recommandation budgétaire:

No concerns.

Please indicate your appraisal of the integration of sex as a biological variable as a strength, weakness, or not applicable to the proposal./Prière de sélectionner une option pour donner votre évaluation de l'intégration du sexe comme variable biologique en tant que point fort ou point faible de la proposition, ou en tant qu'élément non applicable à la proposition.

- (●) **Strength/Point fort**
- ( ) **Weakness/Point faible**
- ( ) **Not applicable/Non applicable**

Please indicate your appraisal of the integration of gender as a socio-cultural determinant of health as a strength, weakness, or not applicable to the proposal./Prière de sélectionner une option pour donner votre évaluation de l'intégration du genre comme déterminant socioculturel de la santé en tant que point fort ou point faible de la proposition, ou en tant qu'élément non applicable à la proposition.

- (●) **Strength/Point fort**
- ( ) **Weakness/Point faible**
- ( ) **Not applicable/Non applicable**

**Review Type / Type d'évaluation:**	Reviewer 1 / Évaluateur 1
**Name of Applicant / Nom du chercheur:**	Schabrun, Siobhan
**Application No. / Numéro de demande:**	517783
**Agency / Agence:**	CIHR/IRSC
**Competition / Concours:**	Project Grant/Subvention Projet
**Committee / Comité:**	Movement & Exercise/Mouvement et exercice
**Title / Titre:**	Unravelling the mechanistic architecture of motor behaviour in pain

Sex and/or Gender Considerations/Notions de sexe et/ou de genre:

Recruitment targets 50:50 M:F enrolment, with n=110 each. This will be enough to test for sex and gender differences and statistical plan is appropriate to account for this.

**Review Type / Type d'évaluation:**	Reviewer 2 / Évaluateur 2
**Name of Applicant / Nom du chercheur:**	Schabrun, Siobhan
**Application No. / Numéro de demande:**	517783
**Agency / Agence:**	CIHR/IRSC
**Competition / Concours:**	Project Grant/Subvention Projet
**Committee / Comité:**	Movement & Exercise/Mouvement et exercice
**Title / Titre:**	Unravelling the mechanistic architecture of motor behaviour in pain

Adjudication Criteria/Critères de sélection

Initial Score/Cote Initiale: 4.5

Top/Bottom Selection/Groupe supérieur/inférieur

- (●) **Top/Groupe supérieur**
- ( ) **Bottom/Groupe inférieur**

**Review Type / Type d'évaluation:**	Reviewer 2 / Évaluateur 2
**Name of Applicant / Nom du chercheur:**	Schabrun, Siobhan
**Application No. / Numéro de demande:**	517783
**Agency / Agence:**	CIHR/IRSC
**Competition / Concours:**	Project Grant/Subvention Projet
**Committee / Comité:**	Movement & Exercise/Mouvement et exercice
**Title / Titre:**	Unravelling the mechanistic architecture of motor behaviour in pain

Summary of Application/Résumé de la demande:

One of the long-term goals of the NPA is to identify mechanisms leading to chronic pain in some people. The main goal of this proposal is to examine if and how cortical mechanisms, and their interaction explain individual variability in motor behaviour in pain.   Their overarching hypothesis is that: While lower sensorimotor cortex excitability and higher GABAergic activity which produce less variable motor behaviours may help management of short-lasting pain by reducing potentially harmful movements, they will eventually lead to more severe, longer-lasting pain if they persist due to reduced sensorimotor integration, central sensitization and a passive cognitive response strategy.

The team has developed methods to measure these variables (variability of motor behavior, sensorimotor integration, central sensitization and cognitive response strategy) and propose to use them as pain develops and subsides in an experimental model of long-lasting pain in humans

**Review Type / Type d'évaluation:**	Reviewer 2 / Évaluateur 2
**Name of Applicant / Nom du chercheur:**	Schabrun, Siobhan
**Application No. / Numéro de demande:**	517783
**Agency / Agence:**	CIHR/IRSC
**Competition / Concours:**	Project Grant/Subvention Projet
**Committee / Comité:**	Movement & Exercise/Mouvement et exercice
**Title / Titre:**	Unravelling the mechanistic architecture of motor behaviour in pain

Strengths and Weaknesses/Forces et faiblesses:

Strengths:

A major strength of this team is the expertise that they have developed for use and validation of the NGF model. To have an experimental model of long-lasting pain in otherwise healthy humans is an invaluable tool to investigate changes that occur with time as pain develops and eventually subsides without confounding factors. The clinical relevance of the model is supported by the similarity of the symptoms observed with those of patients suffering from chronic functional pain, but also by published literature on the involvement of NGF in sensitization. Another strength of the proposal is that the team is experienced with all the proposed measures, and a solid contextual basis and/or preliminary evidence are provided for the use of each of these measures.

The proposal is well written, easy to follow and addresses clearly all issues raised by previous reviewers.

Strong team with highly complementary expertises, all of which are desirable for these experiments, led by highly productive, well-funded mid-career scientists.

Weaknesses

The study will provide large-scale longitudinal data that will be very informative for the use of these methods to evaluate how motor behavior and cortical excitability vary with pain. However, it remains descriptive, and although potential therapeutic strategies guided by these measures (using TMS for instance) are envisaged, there is no proposal to test them even on a small scale for a pilot study.

**Review Type / Type d'évaluation:**	Reviewer 2 / Évaluateur 2
**Name of Applicant / Nom du chercheur:**	Schabrun, Siobhan
**Application No. / Numéro de demande:**	517783
**Agency / Agence:**	CIHR/IRSC
**Competition / Concours:**	Project Grant/Subvention Projet
**Committee / Comité:**	Movement & Exercise/Mouvement et exercice
**Title / Titre:**	Unravelling the mechanistic architecture of motor behaviour in pain

Budget Recommendation/Recommandation budgétaire:

No concerns. Well justified equipment is requested. Is it now allowed on operating grants ?

Please indicate your appraisal of the integration of sex as a biological variable as a strength, weakness, or not applicable to the proposal./Prière de sélectionner une option pour donner votre évaluation de l'intégration du sexe comme variable biologique en tant que point fort ou point faible de la proposition, ou en tant qu'élément non applicable à la proposition.

- (●) **Strength/Point fort**
- ( ) **Weakness/Point faible**
- ( ) **Not applicable/Non applicable**

Please indicate your appraisal of the integration of gender as a socio-cultural determinant of health as a strength, weakness, or not applicable to the proposal./Prière de sélectionner une option pour donner votre évaluation de l'intégration du genre comme déterminant socioculturel de la santé en tant que point fort ou point faible de la proposition, ou en tant qu'élément non applicable à la proposition.

- (●) **Strength/Point fort**
- ( ) **Weakness/Point faible**
- ( ) **Not applicable/Non applicable**

**Review Type / Type d'évaluation:**	Reviewer 2 / Évaluateur 2
**Name of Applicant / Nom du chercheur:**	Schabrun, Siobhan
**Application No. / Numéro de demande:**	517783
**Agency / Agence:**	CIHR/IRSC
**Competition / Concours:**	Project Grant/Subvention Projet
**Committee / Comité:**	Movement & Exercise/Mouvement et exercice
**Title / Titre:**	Unravelling the mechanistic architecture of motor behaviour in pain

Sex and/or Gender Considerations/Notions de sexe et/ou de genre:

Study designed to account for sex (as self reported at birth) and gender differences.

**Review Type / Type d'évaluation:**	Reviewer 3 / Évaluateur 3
**Name of Applicant / Nom du chercheur:**	Schabrun, Siobhan
**Application No. / Numéro de demande:**	517783
**Agency / Agence:**	CIHR/IRSC
**Competition / Concours:**	Project Grant/Subvention Projet
**Committee / Comité:**	Movement & Exercise/Mouvement et exercice
**Title / Titre:**	Unravelling the mechanistic architecture of motor behaviour in pain

Adjudication Criteria/Critères de sélection

Initial Score/Cote Initiale: 4.5

Top/Bottom Selection/Groupe supérieur/inférieur

- (●) **Top/Groupe supérieur**
- ( ) **Bottom/Groupe inférieur**

**Review Type / Type d'évaluation:**	Reviewer 3 / Évaluateur 3
**Name of Applicant / Nom du chercheur:**	Schabrun, Siobhan
**Application No. / Numéro de demande:**	517783
**Agency / Agence:**	CIHR/IRSC
**Competition / Concours:**	Project Grant/Subvention Projet
**Committee / Comité:**	Movement & Exercise/Mouvement et exercice
**Title / Titre:**	Unravelling the mechanistic architecture of motor behaviour in pain

Summary of Application/Résumé de la demande:

This is, I believe, the third submission of this application to the same funding opportunity. The proposal received a high score in the previous round, and the applicants have thoughtfully addressed all concerns raised in the previous review.

The aim of the proposed research is to examine individual differences in cortical mechanisms (sensorimotor cortex excitability, sensorimotor integration, central sensitization and descending pain control, and cognitive response strategy to threat) and motor behavior used in a multidimensional movement task in response to the progressive development, persistence, and resolution of muscle pain.

A longitudinal experimental cohort study will be employed. Pain will be induced in the extensor carpi radialis brevis muscle of 220 healthy individuals using intramuscular injection of nerve growth factor (NGF), an exposure which induces progressively developing, persistent muscle pain. Cortical mechanisms will be assessed i) before pain onset, at days 2 and 4 to evaluate early motor behaviour, at day 8 to evaluate longer-term motor behaviour, at day 21 to examine motor behaviour as pain resolves and at day 40 after pain has resolved and participants have had a period of pain free movement. The primary outcome, described at motor behaviour recorded during the completion of a multidimensional movement task of the wrist. While unclear in the previous submission, the specific outcomes are described as kinematic variability and EMG variability. Growth curve modelling will be used for statistical analyses.

**Review Type / Type d'évaluation:**	Reviewer 3 / Évaluateur 3
**Name of Applicant / Nom du chercheur:**	Schabrun, Siobhan
**Application No. / Numéro de demande:**	517783
**Agency / Agence:**	CIHR/IRSC
**Competition / Concours:**	Project Grant/Subvention Projet
**Committee / Comité:**	Movement & Exercise/Mouvement et exercice
**Title / Titre:**	Unravelling the mechanistic architecture of motor behaviour in pain

Strengths and Weaknesses/Forces et faiblesses:

Strengths:

This is again an extremely well written proposal which aims to understand the mechanisms associated with acute and subacute pain using a novel model (nerve growth factor, NGF) to induce muscle pain which recovers by 21 days.

The NPA has developed and has successfully used NGF model for pain in previous work to elucidate pain physiology. The use of this model is highly valuable in terms of improving our understanding of the link between pain and movement.

The applicants have added a 40 day follow-up to evaluate movement after the pain has resolved.

The team includes three applicants who have demonstrated a strong track record using the proposed methods. The team now also includes a statistician who will supervise an MASc student to undertake the complex statistical models.

Areas for improvement:

The correlation plots presented in Figure 3B to support this proposal based on previous work are still not compelling, however a larger sample will help to clarify the relationship. In particular, figure 3 is said to indicate “an increase in the amplitude of the frontocentral N45 TMS-evoked potential peak in response to short lasting pain”, yet most participants show no change in amplitude with pain, and there is evidence of clustering. It appears that only a small a cluster of participants demonstrate the reported effect rather than there being a meaningful correlation.

While the outcome measures are now described, there is still a lack of clarity around how many outcomes there are. Will kinematic variability is computed in three planes, each analyzed separately? or will there be an overall measure of variability. It appears that there will be at least two outcomes (wrist - flexion, extension, forearm- pronation, supination), but possibly 4 if variability in movement direction is considered separately. Similarly, EMG variability is computed overall or separately for motion in each plane.

While the KT plan remains passive, this is basic science research and thus academic audiences are the predominant knowledge users in this case.

**Review Type / Type d'évaluation:**	Reviewer 3 / Évaluateur 3
**Name of Applicant / Nom du chercheur:**	Schabrun, Siobhan
**Application No. / Numéro de demande:**	517783
**Agency / Agence:**	CIHR/IRSC
**Competition / Concours:**	Project Grant/Subvention Projet
**Committee / Comité:**	Movement & Exercise/Mouvement et exercice
**Title / Titre:**	Unravelling the mechanistic architecture of motor behaviour in pain

Budget Recommendation/Recommandation budgétaire:

Budget: The need for 4 new computers (1 for each trainee) is not adequately justified given the research infrastructure described. The applicants request funds to purchase a TMS compatible EEG system – but do have access to one.

Suggest cut by $100K

**Review Type / Type d'évaluation:**	Reviewer 3 / Évaluateur 3
**Name of Applicant / Nom du chercheur:**	Schabrun, Siobhan
**Application No. / Numéro de demande:**	517783
**Agency / Agence:**	CIHR/IRSC
**Competition / Concours:**	Project Grant/Subvention Projet
**Committee / Comité:**	Movement & Exercise/Mouvement et exercice
**Title / Titre:**	Unravelling the mechanistic architecture of motor behaviour in pain

Please indicate your appraisal of the integration of sex as a biological variable as a strength, weakness, or not applicable to the proposal./Prière de sélectionner une option pour donner votre évaluation de l'intégration du sexe comme variable biologique en tant que point fort ou point faible de la proposition, ou en tant qu'élément non applicable à la proposition.

- (●) **Strength/Point fort**
- ( ) **Weakness/Point faible**
- ( ) **Not applicable/Non applicable**

Please indicate your appraisal of the integration of gender as a socio-cultural determinant of health as a strength, weakness, or not applicable to the proposal./Prière de sélectionner une option pour donner votre évaluation de l'intégration du genre comme déterminant socioculturel de la santé en tant que point fort ou point faible de la proposition, ou en tant qu'élément non applicable à la proposition.

- (●) **Strength/Point fort**
- ( ) **Weakness/Point faible**
- ( ) **Not applicable/Non applicable**

**Review Type / Type d'évaluation:**	Reviewer 3 / Évaluateur 3
**Name of Applicant / Nom du chercheur:**	Schabrun, Siobhan
**Application No. / Numéro de demande:**	517783
**Agency / Agence:**	CIHR/IRSC
**Competition / Concours:**	Project Grant/Subvention Projet
**Committee / Comité:**	Movement & Exercise/Mouvement et exercice
**Title / Titre:**	Unravelling the mechanistic architecture of motor behaviour in pain

Sex and/or Gender Considerations/Notions de sexe et/ou de genre:

well integrated.
